# A systematic scoping review of early interventions for parents of deaf infants

**DOI:** 10.1186/s12887-021-02893-9

**Published:** 2021-10-22

**Authors:** B. Wright, R. Hargate, M. Garside, G. Carr, T. Wakefield, R. Swanwick, I. Noon, P. Simpson

**Affiliations:** 1 Leeds and York Partnership NHS Foundation Trust, COMIC Research, IT Centre, Science Park, University of York, Innovation Way, Heslington, York, YO10 5NP UK; 2 The University College London Ear Institute, 332 Grays Inn Rd, London, WC1X 8EE UK; 3 National Deaf Children’s Society and NatSIP, Ground Floor South, Castle House 37–45 Paul Street, London, EC2A 4LS UK; 4 University of Leeds, School of Education, Hillary Place, Woodhouse, Leeds, LS2 9JT UK; 5 British Association of Teachers of the Deaf, 21, Keating Close, Rochester, ME1 1EQ UK

**Keywords:** Deaf, Early intervention, Newborn Hearing Screening, Parents, Parent support intervention

## Abstract

**Background:**

Over 90% of the 50,000 deaf children in the UK have hearing parents, many of whom were not expecting a deaf child and may require specialist support. Deaf children can experience poorer long-term outcomes than hearing children across a range of domains. After early detection by the Universal Newborn Hearing Screening Programme, parents in the UK receive support from Qualified Teachers of the Deaf and audiologists but resources are tight and intervention support can vary by locality. There are challenges faced due to a lack of clarity around what specific parenting support interventions are most helpful.

**Methods:**

The aim of this research was to complete a systematic scoping review of the evidence to identify early support interventions for parents of deaf infants. From 5577 identified records, 54 met inclusion criteria. Two reviewers screened papers through three rounds before completing data extraction and quality assessment.

**Results:**

Identified parent support interventions included both group and individual sessions in various settings (including online). They were led by a range of professionals and targeted various outcomes. Internationally there were only five randomised controlled trials. Other designs included non-randomised comparison groups, pre / post and other designs e.g. longitudinal, qualitative and case studies. Quality assessment showed few high quality studies with most having some concerns over risk of bias.

**Conclusion:**

Interventions commonly focused on infant language and communication followed by parental knowledge and skills; parent wellbeing and empowerment; and parent/child relationship. There were no interventions that focused specifically on parent support to understand or nurture child socio-emotional development despite this being a well-established area of poor outcome for deaf children. There were few UK studies and research generally was not of high quality. Many studies were not recent and so not in the context of recent healthcare advances. Further research in this area is urgently needed to help develop evidence based early interventions.

**Supplementary Information:**

The online version contains supplementary material available at 10.1186/s12887-021-02893-9.

## Background

### Deaf children and current outcomes

The World Health Organisation (WHO) reports that around 34 million children worldwide are deaf (>40 decibels hearing loss (dBHL) in the better hearing ear) [1]. The Consortium for Research into Deaf Education (CRIDE) survey estimated that there are at least 53,954 deaf (all levels of deafness, from mild to profound) children from birth up to the age of 19 years 11 months in the UK [2]. Over 90% of deaf children in the UK are born to hearing parents, most of whom were not expecting a deaf child [3, 4] and whom may require specialist support and advice.

There is a body of evidence that a significant number of deaf children experience poorer outcomes than hearing children, in terms of educational attainment [5], social domains [6] as well as poorer long term outcomes including increased unemployment [7]. Being deaf can have a range of impacts for both the child and their family [8] including auditory, linguistic, cognitive, social, literacy, and academic functioning [9]. Early language deprivation and language delay can lead to challenges in many developmental domains [10–16] including communication challenges [17, 18], poorer written language [19] and poor academic outcomes [20]. Additionally some deaf children can display difficulties in executive functioning [21], abstract thinking and problem-solving skills that adversely affect academic achievement [22].

### Social emotional development

Language delay [23–25] thought to be associated with language deprivation [26, 27] can in turn lead to delays in Theory of Mind (ToM) (empathy-related) skills [14]. For example, research has shown deaf toddlers exchanging fewer social-communicative signals and having more difficulties understanding the intentions of others [28]. Social and emotional development provides the foundation for how people feel about themselves and experience interactions with others and begins at birth, continuing throughout the lifespan [29]. This unique pattern of social and emotional development may predispose to both serious social challenges and increased psychological distress [15, 30] and are related to a range of other poor socio-emotional outcomes [17, 20, 31].

Problems initiating and sustaining peer relationships and development of self-esteem [32] compound challenges in social settings. Social isolation and low popularity status during childhood have been shown to predict poor emotional well-being in the short term and future adverse consequences for mental health [14, 33]. Deaf children across England not in contact with mental health services have been found to have 2 to 3 times the rates of mental health problems compared to other children [34] and a systematic review found higher rates of behaviour problems in deaf children across numerous countries [35].

### Current international guidelines

The Universal Newborn Hearing Screening Programme (UNHSP) was introduced across the UK between 2002 and 2006 [36] to screen all infants shortly after birth for signs that they may be deaf. Results to date suggest positive associated language [37] and health economic [38] outcomes. Early detection followed by specialist support programmes have been found to improve many negative outcomes for deaf children [39–41] including language acquisition and learning skills [29, 42, 43]. However, the outcomes for deaf children are highly variable due to many different factors including those described above [44].

On learning that their child is deaf, parents require a level of emotional support to adjust to this new information during the transition from diagnosis to early management [45, 46]. Families must navigate through a myriad of services and choices including the NHS, local authorities and private agencies, and their own family constructs, all of which requires sensitivity to the social and emotional needs of a family with a deaf baby [47]. A consensus statement on early intervention lays out aspirations for good practice [48]. Current guidance is that early intervention for deaf infants should include parents as the most important agents for supporting their young children’s language development [16] and that professionals who work with parents should focus on promoting their abilities to provide a language-rich environment [42, 48, 49]. Family-centred care has been advocated as the optimal way of addressing family needs in early intervention [50].

Most countries provide a range of support to families during early hearing detection and intervention programs. In the US parents receive information from an audiologist, written information and discussion with a medical professional [50]. Various states offer different additional support. For example in Colorado, USA all families of diagnosed children are referred to an early intervention system that begins with a counselling and information session with a deaf early intervention provider. Subsequently an early intervention co-ordinator consults with the family, establishes links to the local education pathways, offers access to sign language training for the family and engages them in a 6 month state wide program of support. This is offered to all families. However there has been no randomised controlled trial (RCT) to evaluate this to date, and no other published studies which met our PICOS criteria and so it does not appear in this review. In the UK Qualified Teachers of the Deaf (QToD) play an important role in providing early education services to families of infants and toddlers with hearing loss [51]. There is an element of universal provision but these services are often needs led depending on factors including audiological evaluation, child factors such as other significant medical conditions, social factors and parental choice. Current protocol in England is that every child identified by the UNHSP is contacted by a QToD within two working days post-referral [52]. The QToD works closely with the parent after the initial meeting and diagnostic period. This support is ongoing during the pre-school period. Formal parent support interventions are not often used but the regular QToD support is holistic, not just concentrating on language and communication but also on social and emotional development, audiological support and signposting to additional services if necessary. Many services will use the National Sensory Impairment Partnership (NatSIP) Eligibility Framework [53] and their specific early years’ guidelines [54, 55] to determine levels of support according to individual need. This framework includes weighting of specific criteria but it is clear there still needs to be a focus on individual needs. However, provision is not universal, leading to variable offers for parents in different localities which may lead to deaf babies and young children of similar need getting inconsistent support due to a lack of resources despite the best efforts of specialist services. One study in Australia has reported such a challenge with many gaps in family-centred service provision at the time of identification and after enrolment in early hearing intervention services [18].

Many UK services have an Early Years (EYs) specialist QToD who works with these families at this stage. They may follow the Early Support Monitoring Protocol (revised from September 2020 as Success from the Start) but this is not mandatory, nor is it a formal intervention programme but a developmental resource used mainly to work with families to support them to observe, monitor and record progress their children make. QToDs receive some early years guidance during their training, delivered within the full training protocol.

### Barriers of access to support and eligibility criteria

Even where intervention is available, families are extremely diverse and several factors may hinder families from accessing early intervention. These include language barriers with parents, lack of availability as well as cultural challenges during audiological testing (e.g., families sometimes preferred male practitioners, cultures where disability is stigmatised sometimes leading to caregivers denying the hearing loss or declining support or amplification for their children) [56].

Based on an ever-growing body of research, family-centred practices are a recommended, evidence-based principle of early childhood intervention but there continues to be a gap between recommendations and implementation of such practice across all disciplines [57].

### What are the gaps in the literature that we need to fill?

A comprehensive synthesis of the available research evidence is necessary to inform a discussion about appropriate provision of support and interventions for parents of deaf infants. A well-established key criterion for the implementation of screening in society is the existence of a helpful and accepted intervention [58] which in the context of new-born hearing screening is early intervention with parents [48, 59].

## Methods

The main aim of this review was to identify the available literature for early parenting interventions for deaf infants.

This research seeks to add to the evidence base by providing an up to date systematic scoping review of parent support interventions for deaf infants, synthesising the targets of these interventions and highlighting any evidence gaps to inform subsequent early intervention training and research in a UK context.

The review was registered on the PROSPERO database: CRD42019138001.

### Stakeholder workshops

Prior to running literature searches the study team held national workshops with service providers and academics to gather information about the support that services currently offer parents of deaf infants aged 0-5 years old. The workshops included presentations and group discussions about good practice, the eligibility criteria for access to interventions, barriers to practice and gaps in research. The research team also held a meeting with parents of deaf infants to gather information about their experiences of the support they have been offered. Notes from discussion at these workshops and meetings were collated and key intervention elements and outcomes were identified, and included in the comprehensive search strategy for the systematic scoping review.

### Systematic scoping review

Key stakeholders were identified from the workshops and invited to form an expert panel. The search strategy and PICOS criteria were developed with input from the workshops and finalised with the expert panel.

The final PICOS were as follows:

#### Population

- Deaf and/or hearing parents with children identified as deaf aged between birth and 5 years
- Children who are mild to profoundly deaf (between 40dB to 140dB on the audiogram in both ears) regardless of whether they are sign language users, or use hearing aids, have received cochlear implantation or use other communication aids

#### Intervention

- Any parenting intervention and/or support for this population

For example; support for parents, parenting groups, skills training, provision of information, therapy groups, family therapy or language and communication support in a family context or delivered through the parent

- Any setting (e.g. home based, school, health centre, hospital) including both group and 1:1 interventions, with or without the involvement of the child

#### Comparison

- Where the study is a randomised controlled trial we included any comparator (control conditions or other active comparators)
- Other designs were included and we applied the same comparator criteria for any study using a comparison group (e.g. non-randomised controlled designs)
- Other empirical designs such as pre-post designs were included in the absence of a comparator

#### Outcomes

- Child and parent outcomes such as language acquisition and communicative development, socio-emotional outcomes (including mental health outcomes) and cognitive and educational outcomes
- Behavioural, quality of life outcomes or developmental milestones, as well as parenting outcomes such as studies measuring parental emotions, language, communication, stress and mental wellbeing
- Validated instruments were preferred where possible; however, studies measuring descriptive statistics were included where relevant (e.g. vocabulary obtained or educational outcomes)

#### Study types

- We considered all study designs (e.g. randomised and non-randomised intervention studies and observational studies including cohort, qualitative evaluations, descriptive, case-control, cross-sectional)
- Studies could be conducted in any country and no exclusions were made on the basis of publication status, date or language, we explored processes for translation where necessary. If conference abstracts were identified, they were included if they provided enough information for data extraction. If they did not, details were sought from the author or an available results paper.

### Search strategy

A search strategy was designed to capture the population of parents/caregivers of children diagnosed as deaf and a range of interventions (based on existing knowledge and feedback from the stakeholder workshops). A full search strategy can be found in Additional file 1.

Searches were loaded into EndNote bibliographic software and then transferred to an Excel database for sifting. The searches were run by an information specialist at the Centre for Reviews and Dissemination (CRD, University of York) on the following databases in September 2019: CINAHL, Cochrane Central Register of Controlled Trials (CENTRAL), Embase, MEDLINE, PsycINFO, Science Citation Index, Scopus and Social Science Citation Index.

### Screening and data extraction

Two independent reviewers conducted screening over three sifting stages. References of reviews, books, and included literature were checked to ensure all relevant papers were identified. Agreement for each sift was calculated over a minimum 10% overlap, and was consistently above the pre-specified 80%. Disagreements were discussed and resolved with a third independent reviewer. Authors were contacted where necessary to clarify outcome measures and results.

For each eligible study, data was extracted by two reviewers using the Template for Intervention Description and Replication (TIDieR) checklist [60] as a guideline for descriptions of included interventions. The two reviewers conducted quality assessment using the Revised Cochrane Collaborations Assessment Tool for Assessing Risk of Bias in Randomised Trials (ROB-2) [61] tool for RCTs and the Risk Of Bias In Non-Randomised Studies of Interventions tool (ROBINS-I) [62] for non-randomised studies (comparison groups and pre-post study designs). Additional file 2 includes a table (Table 1) of characteristics for included studies in the review. This presents further detail on the data extracted.

### Synthesis

For randomised controlled trial studies only, we compared these to the elements outlined in the International Consensus Statement [48] to determine how many of these 10 principles of best practise were included.

The final included studies were discussed at an expert panel meeting. At this meeting we discussed the most appropriate way to present the studies as well as the key outcomes to share. As a result, themes were identified, based on the primary focus of the intervention, and these have been used to summarise the studies.

## Results

**Fig. 1:**
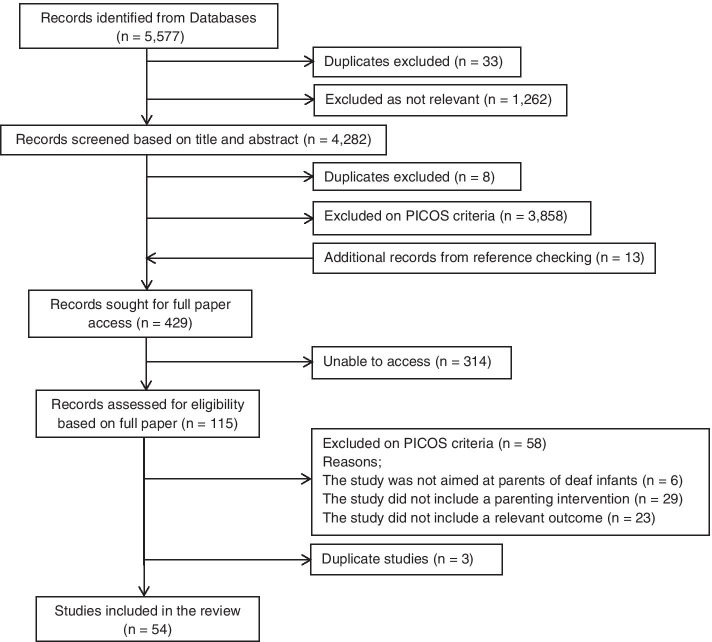
PRISMA Diagram

5577 records were identified through database searches. The review was completed by two independent reviewers over three sifts. The first two sifts examined records based on title and abstract. 41 were excluded by reviewers as duplications, 1,262 were identified as not relevant and 3,858 were excluded as they did not meet the PICOS criteria (see Fig. 1 for CONSORT). 13 additional records were added through reference checking. 429 papers were sought for full paper screening. If papers could not be accessed by the research team, we contacted relevant library services and the authors where necessary to obtain these. We had discussions with two authors to ensure we had captured relevant papers. There remained a number of papers which could not be accessed (n = 314), potentially due to the lack of restriction on date. Reviewers conducted extensive reference checking to ensure no relevant papers were missed. 115 papers were included for full paper screening. 58 papers were excluded at this full paper third sift and a further 3 papers were excluded as duplicates. This left 54 included papers for data extraction.

Included study designs consisted of 5 randomised controlled trials, 17 non randomised comparison groups, 12 pre / post and 20 other designs (including longitudinal, qualitative evaluations, sub-analyses, file reviews, case studies).

Of those where the design enabled quality assessment (34 of the 54), 1 was deemed high-risk of bias, 4 had some concerns of bias, 18 were moderate risk, 10 low risk and one did not have sufficient detail to assess. For the remaining 20 studies, their designs did not allow for meaningful quality assessment to be conducted. Commonly identified issues included; lack of blinding, differences between groups at baseline (that was not controlled for), measures included self-report or observations and a lack of information provided on processes used for recruiting and allocating participants to groups.

Research outcomes were grouped by themes based on the primary focus of the intervention. Where there was an overlap between themes, the main outcome measures were considered to help categorise the studies. Four themes were identified; language and communication, parental wellbeing and empowerment, parental knowledge and skill and parent child relationship. These are discussed in further detail later.

The results are summarised in Table 1 (Table 1. Summary Results Table by Study Design) broken down by methodology, risk of bias and intervention focus.

**Table 1:** Summary results table by study Design

	Total Number	Risk of Bias	Theme
**RCTs**	5	4 some concerns 1 high risk	2 language and communication 3 parent wellbeing and empowerment
**Comparison Group**	17	14 moderate 2 low 1 not enough information provided	9 language and communication 2 parent wellbeing and empowerment 4 parent knowledge and skills 2 parent child relationship
**Pre-Post**	12	4 moderate 8 low	7 language and communication 3 parent wellbeing and empowerment 1 parental knowledge and skills 1 parent child relationship
**Others**	20	N/A	11 language and communication 1 parent wellbeing and empowerment 8 parental knowledge and skills
**Total**	54	1 high risk 22 moderate / some concerns 10 low 1 not enough information provided 20 N/A	29 language and communication 9 parent wellbeing and empowerment 13 parental knowledge and skills 3 parent and child relationship

The most common target of intervention was language and communication (29 of the 54 studies). All 29 interventions in this category were found to have an effect, 17 of which were deemed significant although it is important to note methodological quality varied greatly.

13 interventions focused on parental knowledge and skills although there were several overlaps between this theme and the themes of parental empowerment and parent – child relationship. Intervention focus areas included skills for shared reading with the child (5 of the 13 interventions), techniques for managing child behaviour and hearing aid inspection.

9 interventions focused on parental wellbeing and empowerment. Parental wellbeing interventions largely looked at reducing parental stress and / or increasing parental support. Parental empowerment was often centred on decision making and accessing appropriate intervention as a way to reduce parental needs.

Only 3 interventions specifically focused on the parent child relationship. Two of these interventions found video feedback to be effective at increasing parental emotional availability although the results were not significant.

### Randomised Controlled Trials (RCTs)

The following interventions were found to have a significant effect in randomised controlled trials:

#### Parent-implemented communication treatment (PICT)

This intervention includes strategies (visual, interactive, tactile and linguistic stimulation) to promote early communication (all forms including vocal, gestures and non-verbal) and parental sensitivity in everyday routines. In this study, parents in the treatment group increased their use of communication support strategies by 17% (effect size 1.08, p = .04). For children, larger gains were seen in pre-linguistic skills in treatment groups compared to control groups (effect size of 1.09, p = .03) [63].

#### Muenster Parenting Programme

This programme focuses on enhancing parents’ responsive communicative behaviour to vocal and non-verbal signals of the child, and to reduce inappropriate parental behaviour that is too strongly initiative (e.g. inadequate introduction of a new action/object, neglecting the child’s current focus of attention) [64]. There were significant increases in pre-post scores for trained parents in their responsiveness and a reduction in inappropriate initiative behaviour but not for the control group.

#### Educational intervention program based on empowerment of mothers

A training package was developed aiming to empower parents, by increasing their knowledge of interventions and improving their self-confidence [65]. This intervention focused on empowerment of parents, and also collected outcomes based on child speech development. Significant differences were seen in the intervention group’s pre-post test scores for mothers' empowerment, self-efficacy, and adaptation. There was also a significant reduction for children’s scores on the Newsha Developmental Speech Scale indicating a reduction in the severity of speech disorders.

The final two RCTs tested ‘Baby Portal’ (a social network to facilitate discussion between parents) [66] and a ‘Self-Instructional Parenting Program’ (addressing feelings, attitudes and perceptions of interactions and teaching behaviour management principles to reduce stress) [67]. Neither found significant differences for those accessing the interventions.

### International Consensus Statement

There is currently a ‘Best Practices in Family-Centered Early Intervention for Children Who Are Deaf or Hard of Hearing: An International Consensus Statement’ [48] of 10 principles which are deemed to be best practice for early intervention in this population. We mapped the interventions with RCT evidence to these 10 principles: however we found that none of the 5 RCTs included all 10 of the consensus principles. Commonly included was Principle 4: Family Social and Emotional Support, whereby families were provided with support systems to help them access knowledge and experience, and Principle 7: Qualified Providers, where those providing services to families had the necessary knowledge and experience. The included RCTs were less likely to report on Principle 3: Informed choice and decision making, relating to interventions where services provide families with the knowledge to make decisions based on special education laws and their rights and Principle 9: Progress Monitoring, relating to regular assessments of progress throughout the intervention. However, when comparing to the 10 consensus principles, we were only able to use available information about the intervention as provided in the papers, which were often limited in detail.

### Non-randomised or controlled group designs

There were 9 studies focused on language and communication, 6 of which found a significant effect. These included studies which aimed to teach caregivers strategies to improve use of sign language and child vocabulary such as Adult "recasting" in sign language [68] and the ASPIRE Intervention Curriculum [69]. Auditory Verbal (AV) therapy [70] was also found to significantly improve language development in children using hearing technologies, and the Tri sensory language stimulation [71] found significant effects on nine out of nineteen variables related to language and vocabulary learning. Other studies included comprehensive training programmes, such as Bill Wilkerson Hearing and Speech Centre Program [72]. They found that children receiving early intervention had similar language competence to that of hearing children, and performed significantly better than those receiving late intervention. The Counselling and Home Training Program for Deaf Children [73] is a family oriented programme that encourages natural communication and building competence and esteem in a secure family context. The 1984 publication focuses on communication outcomes, with significantly more developmentally mature communication and high interaction in families who had received the intervention.

There were 3 studies which showed non-significant improvements from the intervention on the child’s language and communication; these include the Tracy Clinic Oral Preschool [74]; a Parent Orientation Program [75] which focuses on early exposure to manual communication and The SKI*HI program [76] which teaches parents how to provide auditory and language stimulation for the child in the home.

4 studies focused on parental knowledge and skills, with significant results for a second study of PCIT [77] where reported outcomes focused on parent skills and managing child behaviour. There were also significant results for Interactive Storybook Reading [78], where the main outcomes focused on parent behavior, engagement, teacher techniques, and interactive reading. 2 studies showed non-significant improvements; the Deaf Mentorship Programme [79] where deaf adult mentors visit families to share their language, culture, and personal knowledge and the Parent Study Group [80] which is based on the Adlerian Model and helps parents manage their child’s behaviour.

Both of the studies focused on parent wellbeing and empowerment showed significant results. These include The Counselling and Home Training Program for Deaf Children [81] which reports reduced parenting stress, and the family focused early intervention group [82] which focuses on home training for families in rural areas.

Both of the studies that focused on parent child relationships showed non-significant improvements in parental scores on the Emotional Availability Scales: Psychosocial video intervention, [83] and on the Gerrard parent-child questionnaire: Faranak parent-child program, [84].

### Pre/post study designs

7 studies focused on language and communication, four of which showed significant results for increases in parent facilitation of spoken language: Parental Training Course, [85]; parent conversational ability Hanen based training program, [86]; child vocalisations ASPIRE intervention, [87]; and child hearing and speech skills Parent counselling, [88]. The other three studies showed non-significant improvements in learning and expression of language: Total communication, [89] and gains in child vocabulary SSE-2, [90]; Parent and child training, [91].

Of the 3 studies focused on parent wellbeing and empowerment, a group counselling programme showed significant improvements for parents: Parent Group Counselling Programme [92]. A remote intervention delivery service found no difference between tele intervention and conventional intervention in terms of communication performance of children meaning that telecommunication could be used as effectively as face to face; Tele intervention [93]. There were non-significant improvements for families in the HI HOPES [94] intervention, which has the central aim of informing and equipping parents to make their own decisions and measured parent satisfaction and child language outcomes.

The remaining 2 pre/post study designs focused on parental knowledge and skills, with a storybook reading programme reporting non-significant improvements: Parent child reading training [95] and on the parent child relationship, with a video feedback intervention showing non-significant maintenance and improvement on parent Emotional Availability scores: Video Interaction Guidance (VIG) [96].

### Other study designs

For those studies that used other study designs, 11 focused on language and communication. Hogan et al. [97] predicted language scores based off modelling for Auditory-Verbal Therapy, but found no significant difference in rate of language development. Calderon and Low [98] conducted a sub-analysis of a larger study of Early Childhood Home Instruction and found non-significant improvements in language when a father was present at the intervention. Three studies looked at language communication using a longitudinal design; Oral Language Training [99]; Central Institute for the Deaf Early Education Project [100]; A Good Future for Deaf Children Programme [101], and all found improvements in language and communication for the child. There were also 6 papers that reported results from either case or file review, or case studies, all reporting improvements for the child’s language; Auditory-Verbal Therapy [102]; The 10-year-old Early Childhood Home Instruction Program for Hearing-Impaired Infants and Their Families [103]; PiCS Intervention using distance education technology [104]; A program for teaching written language [105]; HI CHIPS Total Communication Program [106]; Communication Program [107].

8 studies focused on parental knowledge and skills including one logic model evaluation testing The Shared Reading Project [108]. There were 5 case studies, 3 showing positive changes for families; Parent Hearing Aid check training [109]; Educator-oriented contact intervention program [110]; Treatment Program [111]; one showing highly mixed results; Iowa E-Book [112], and one showing the intervention to be not effective at changing parent behaviour: Parent Training on Storybook Reading [113].

One study focused on parent wellbeing and empowerment using a retrospective survey design; Diagnostic Early Intervention Program (DEIP) [114], with findings showing family involvement and early enrolment as important factors for improving child outcomes.

The remaining 2 studies based on parental knowledge and skills were qualitative; Family-oriented early intervention program [115]; ASL Parent-Child Mother Goose [116], with both finding positive impacts for mothers.

## Discussion

In summary, we found 5 RCTs which focussed mainly on parental wellbeing and empowerment (n=3) or language and communication (n=2). No RCTs were identified which tested interventions targeting the social and emotional development of the child. The categorisations of the themes of the studies are to be interpreted with caution, as there does remain overlap, with some papers fitting more than one theme. For example, some interventions describe the main aim of the programme to empower the parent in making decisions, but the main outcomes measured relate to child language development. The research team has aimed to group them based on the overarching intervention theme from the paper, to give the most meaningful overview of the current literature base in this field (as well as providing some type of comparison between a high number of included papers, which encompass a broad range of outcome measures and focuses). The large number of included studies was due to the PICOS criteria used being broad and very inclusive of a range of study parameters. This was intentional as the aim was to be able to provide a comprehensive overview of the current literature and be able to identify any gaps in research.

The largest gaps in the research appear to be threefold. Firstly there are only 5 randomised controlled trials of early parent interventions for deaf infants. Given that there are 34 million deaf children internationally, 5 RCTs is a remarkably small number of studies. Whilst there are some ethical challenges to carrying out RCTs in vulnerable populations this highlights the fact that we have little understanding of what kind of specialist support interventions can best improve outcomes for deaf infants.

Secondly the focus of the majority of the included interventions (29 of the 54) was on the language and communication development of the child or parental well-being, rather than wider aspects of the complex development and neurodevelopment of deaf children including for example their social and emotional development [117]. Language and communication is likely to be at the root of many of these difficulties particularly where infants have limited exposure to language learning [26, 27, 118]. The focus on language and communication is important but can lead parents to create a range of expectations, hopes and stressors related to this one area of development [119], and may have an impact on the way parents, families and professionals are enabled to have discussions about holistic aspects of quality of life. It may also inadvertently divert attention away from a range of other important aspects of development such as social and emotional development. The focus on language may also affect family stress and child outcomes. Indeed researchers reporting on language and literacy outcomes after the introduction of the UNHSP [120] have called for a broader range of outcomes to be attached to it [121] in order to better understand the broad range of developmental impacts on deaf children and their families. It would be a recommendation that current guidelines in this field, such as the International Guidelines for Best Practice [48] also emphasise a holistic approach, with a strong focus on social and emotional development of the child.

Thirdly, to scope the widest range of possible interventions the included literature spans from 1970 to 2019, with 12 published in the last 5 years. This mean that many of the programmes are less current and were conducted before the introduction of UNHSP, with very few examining issues of early intervention in terms of content or effect. The broad range of programmes also spans across cultural contexts [122] and so this may affect the way they are conceptualised, implemented, received and assessed, leading to a lack of generalisability. Many of the studies focused on short term outcomes, with few following outcomes in the medium to long term which would be useful to assess in any future research. There were also concerns over risk of bias in many of the studies assessed.

## Conclusion

Training is necessary for support staff to be responsive to families’ unique and often complex needs [123–125]. While UK QToDs receive guidance during training for working with early years children this is relatively limited, delivered within the full training protocol and centred around child development and language acquisition with the main focus of the course being centred around school aged children. Previously in the UK an additional qualification (Early Years ToDs PGDip/MA) in working with deaf babies under the age of two was available delivered by University of Hertfordshire/Mary Hare and many services have an Early Years (EYs) specialist QToD. The full course has now closed but it is still possible to complete individual modules. There is some very important work that needs to be done to understand what training is required, what parent support works well for different children in specific circumstances, how and when it should be delivered and to explore what evidence-based support interventions should be universally offered and what support is tailored to the individual needs of children and families. It is important to increase the availability of properly funded evidence based interventions and training opportunities to ensure that ToDs have a consistent approach to be able to provide individualised and responsive support to meet the widely varying needs of deaf children and their families.

The Joint Committee on Infant Hearing (JCIH) Position Statement 2007 expresses the goal of new-born hearing screening as ‘to maximise linguistic competence and literacy development for children who are deaf or hard of hearing’. An international consensus statement has been released describing the principles for Family-Centered Early Intervention for Children Who Are Deaf or Hard of Hearing (of 10 principles) which are deemed to be of best practice for early intervention in this population [48, 126, 127]. One potential gap in these statements is the relatively sparse mention of socio-emotional development and needs of deaf children.

The parent child relationship is important for the social and emotional development of deaf children [48] and further research could usefully explore how this may mediate outcomes and how it may be enhanced, including support for parents. Future research could also usefully include a wider set outcomes (alongside language development) such as theory of mind, empathy and executive functioning development, and collection of long term outcomes to reflect this. Since much of this research is before the advent of the UNHSP more current research is urgently needed.

## Supplementary Information

**Additional file 1. **Full search strategy.

**Additional file 2: Table 1**: Characteristics of Included Studies.

## Data Availability

All data generated or analysed during this study are included in this published article

## Abbreviations

CRIDE: Consortium for Research into Deaf Education
CRD: Centre for Reviews and Dissemination
dBHL: Decibels Hearing Loss
DEIP: Diagnostic Early Intervention Program
EYs: Early Years
JCIH: Joint Committee on Infant Hearing
NatSIP: National Sensory Impairment Partnership
PICT: Parent-implemented communication treatment
QToD: Qualified Teachers of the Deaf
RCTs: Randomised Controlled Trials
ROBINS-I: Risk Of Bias In Non-Randomised Studies of Interventions
TIDieR: Template for Intervention Description and Replication
ToM: Theory of Mind
UNHSP: Universal Newborn Hearing Screening Programme
WHO: World Health Organisation
