# Supplementary material for: A systematic scoping review of early interventions for parents of deaf infants
Source: BMC Pediatr. 2021 Oct 22;21:467. doi: 10.1186/s12887-021-02893-9 (PMC8532316; doi:10.1186/s12887-021-02893-9)

**Parental interventions and deaf children**

**Literature searching: 20^th^ September 2019**

The following bibliographic databases were searched:

CINAHL, Cochrane Central Register of Controlled Trials (CENTRAL), Embase, MEDLINE, PsycINFO, Science Citation Index, Scopus and Social Science Citation Index

and a total of 9062 records were identified. These were loaded into EndNote bibliographic software and deduplicated to leave a total of 5577 unique records.

Each of the search strategies used is reproduced below, with details of the search date, interface used and number of records retrieved

**Cochrane Central Register of Controlled Trials via John Wiley’s Cochrane Library**

**Search date 18^th^ September 2019**

**Records identified=72**

#1 MeSH descriptor: [Child] explode all trees

#2 MeSH descriptor: [Infant] explode all trees

#3 ((child* or infant* or infancy or preschool* or "pre school*" or baby or babies or pediat* or paediat*)):ti,ab,kw (Word variations have been searched)

#4 (boy or boys or girl or girls or youngster*):ti,ab,kw

#5 #1 OR #2 OR #3 OR #4

#6 MeSH descriptor: [Persons With Hearing Impairments] explode all trees

#7 MeSH descriptor: [Hearing Disorders] explode all trees

#8 MeSH descriptor: [Deaf-Blind Disorders] explode all trees

#9 #6 OR #7 OR #8

#10 #5 AND #9

#11 (child* or infant* or infancy or preschool* or "pre school*" or baby or babies or pediat* or paediat*) NEAR/4 (deaf* or hearing* or hard-of-hearing or listen*)

#12 #10 or #11

#13 MeSH descriptor: [Parents] explode all trees

#14 MeSH descriptor: [Parent-Child Relations] explode all trees

#15 MeSH descriptor: [Caregivers] explode all trees

#16 MeSH descriptor: [Parenting] explode all trees

#17 #13 or #14 or #15 or #16

#18 #12 and #17

#19 (parent* or mother* or maternal* or mum* or father* or paternal* or dad* or carer* or caregiver*) near/3 intervention*

#20 (parent* or mother* or maternal* or mum* or father* or paternal* or dad* or carer* or caregiver*) near/3 (advice* or advisory or counsel* or helpline* or information* or pack* or signpost* or support* or telephone* or therap* or visit*)

#21 (parent* or mother* or maternal* or mum* or father* or paternal* or dad* or carer* or caregiver*) near/3 (advisor* or audiolog* or consultant* or instructor* or psychologist* or teacher* or technician* or therapist*)

#22 (parent* or mother* or maternal* or mum* or father* or paternal* or dad* or carer* or caregiver*) near/3 (email* or facebook* or internet* or media* or online* or website* or whatsapp)

#23 (parent* or mother* or maternal* or mum* or father* or paternal* or dad* or carer* or caregiver*) near/3 (course* or drop* or drop-in or group* or home* or program* or represent* or train* or workshop*)

#24 #19 or #20 or #21 or #22 or #23

#25 #12 and #24

#26 "deaf cafe"

#27 teacher* near/3 deaf*

#28 "cued speech"

#29 "british sign language" near/2 (class* or lesson*)

#30 BSL near/2 (class* or lesson*)

#31 "sign language" near/2 (class* or lesson*)

#32 "family sign language" near/2 (class* or lesson*)

#33 "deaf role model*"

#34 "baby beat*"

#35 "deaf instructor*"

#36 "sign and play"

#37 "sing and sign"

#38 marschak

#39 theraplay

#40 "filial therapy"

#41 "auditory verbal therapy"

#42 #26 OR #27 OR #28 OR #29 OR #30 OR #31 OR #32 OR #33 OR #34 OR #35 OR #36 OR #37 OR #38 OR #39 OR #40 OR #41

#43 #18 OR #25 OR #42

**CINAHL Complete**

**Via EBSCO**

**Search date= 16^th^ September 2019**

**Records identified=710**

| (MH "Child+") | **Expanders** - Apply equivalent subjects  **Search modes** - Boolean/Phrase |  |
| --- | --- | --- |
| S2 | (MH "Infant+") | (227,722) |
| S3 | TI ( child* or infant* or infancy or preschool* or pre school* or baby or babies or pediat* or paediat* ) OR AB ( child* or infant* or infancy or preschool* or pre school* or baby or babies or pediat* or paediat* ) OR TI ( boy or boys or girl or girls or youngster* ) OR AB ( boy or boys or girl or girls or youngster* ) | (582,312) |
| S4 | S1 OR S2 OR S3 | (802,219) |
| S5 | (MH "Deaf-Blind Disorders+") | (395) |
| S6 | (MH "Hearing Loss, Functional") OR (MH "Hearing Loss, Partial") OR (MH "Hearing Loss, Conductive") OR (MH "Hearing Loss, High-Frequency") OR (MH "Hearing Loss, Noise-Induced") OR (MH "Hearing Loss, Sensorineural+") | (8,834) |
| S7 | (MH "Rehabilitation of Hearing Impaired+") | (4,888) |
| S8 | S5 OR S6 OR S7 | (13,635) |
| S9 | S4 AND S8 | (4,060) |
| S10 | TI ( (child* or infant* or infancy or preschool* or pre school* or baby or babies or pediat* or paediat*) N2 (deaf*or hearing* or hard-of-hearing or listening-impair*) ) OR AB ( (child* or infant* or infancy or preschool* or pre school* or baby or babies or pediat* or paediat*) N2 (deaf*or hearing* or hard-of-hearing or listening-impair*) ) | (236) |
| S11 | S9 OR S10 | (4,235) |
| S12 | (MH "Parents of Disabled Children") OR (MH "Parents") | (42,362) |
| S13 | (MH "Parenting") | (15,481) |
| S14 | (MH "Caregivers") | (30,964) |
| S15 | (MH "Parent-Child Relations") | (17,761) |
| S16 | S12 OR S13 OR S14 OR S15 | (94,914) |
| S17 | S11 AND S16 | (285) |
| S18 | TI ( (parent* or mother* or maternal* or mum* or father* or paternal* or dad* or carer* or caregiver*) N3 intervention* ) OR AB ( (parent* or mother* or maternal* or mum* or father* or paternal* or dad* or carer* or caregiver*) N3 intervention* ) OR TI ( (parent* or mother* or maternal* or mum* or father* or paternal* or dad* or carer* or caregiver*) N3 (advice* or advisory or counsel* or helpline* or information* or pack* or signpost* or support* or telephone* or therap* or visit*) ) OR AB ( (p [...](javascript:showHistoryTerm('ctl00_ctl00_FindField_FindField_historyControl_HistoryRepeater_ctl17_ellipsis',true)) | (34,458) |
| S19 | TI ( (parent* or mother* or maternal* or mum* or father* or paternal* or dad* or carer* or caregiver*) N3 (advisor* or audiolog* or consultant* or instructor* or psychologist* or teacher* or technician* or therapist*) ) OR AB ( (parent* or mother* or maternal* or mum* or father* or paternal* or dad* or carer* or caregiver*) N3 (advisor* or audiolog* or consultant* or instructor* or psychologist* or teacher* or technician* or therapist*) ) OR TI ( (parent* or mother* or maternal* or mum* or fathe [...](javascript:showHistoryTerm('ctl00_ctl00_FindField_FindField_historyControl_HistoryRepeater_ctl18_ellipsis',true)) | (33,555) |
| S20 | S18 OR S19 | (58,649) |
| S21 | S11 AND S20 | (165) |
| S22 | S17 OR S21 | (373) |
| S23 | TI "deaf cafe" OR TI teacher* N3 deaf* OR TI "cued speech" OR TI ( (british sign language) N2 (class* or lesson*) ) OR TI ( BSL N2 (class* or lesson*) ) OR TI ( (sign language) N2 (class* or lesson*) ) OR TI "deaf role model*" OR TI baby beat* OR TI "deaf instructor*" OR TI ( "sign and play" ) OR TI ( "sign and sing" ) OR TI marschak | (130) |
| S24 | AB "deaf cafe" OR teacher* N3 deaf* OR "cued speech" OR ( (british sign language) N2 (class* or lesson*) ) OR ( BSL N2 (class* or lesson*) ) OR ( (sign language) N2 (class* or lesson*) ) OR "deaf role model*" OR baby beat* OR "deaf instructor*" OR ( "sign and play" ) OR ( "sign and sing" ) OR marschak | (205) |
| S25 | TI ( theraplay OR "filial therapy" OR "auditory verbal therapy" ) OR AB ( theraplay OR "filial therapy" OR "auditory verbal therapy" ) | (77) |
| S26 | S23 OR S24 OR S25 | (350) |
| S27 | S22 OR S26 | (710) |

**Embase**

**Via OVID**

**Search date=16/08/2019**

**Records identified=1389**

**Database: Embase <1974 to 2019 September 13>**

1 exp Child/ (2500758)

2 exp Infant/ (947764)

3 (child$ or infant$ or infancy or preschool$ or pre school$ or baby or babies or pediat$ or paediat$).ti,ab. (2209781)

4 (boy or boys or girl or girls or youngster$).ti,ab. (293500)

5 1 or 2 or 3 or 4 (3242145)

6 exp Hearing Impairment/ (90845)

7 exp Hearing Disorder/ (123017)

8 Deafblindness/ (164)

9 6 or 7 or 8 (123017)

10 5 and 9 (35161)

11 ((child$ or infant$ or infancy or preschool$ or pre school$ or baby or babies or pediat$ or paediat$) adj4 (deaf$ or hearing$ or hard-of-hearing or listen$)).ti,ab. (14794)

12 10 or 11 (39882)

13 exp Parent/ (231226)

14 Child Parent Relation/ (49442)

15 exp Caregiver/ (74484)

16 13 or 14 or 15 (333139)

17 12 and 16 (2238)

18 ((parent$ or mother$ or maternal$ or mum$ or father$ or paternal$ or dad$ or carer$ or caregiver$) adj3 intervention$).ti,ab. (13097)

19 ((parent$ or mother$ or maternal$ or mum$ or father$ or paternal$ or dad$ or carer$ or caregiver$) adj3 (advice$ or advisory or counsel$ or helpline$ or information$ or pack$ or signpost$ or support$ or telephone$ or therap$ or visit$)).ti,ab. (53086)

20 ((parent$ or mother$ or maternal$ or mum$ or father$ or paternal$ or dad$ or carer$ or caregiver$) adj3 (advisor$ or audiolog$ or consultant$ or instructor$ or psychologist$ or teacher$ or technician$ or therapist$)).ti,ab. (10708)

21 ((parent$ or mother$ or maternal$ or mum$ or father$ or paternal$ or dad$ or carer$ or caregiver$) adj3 (email$ or facebook$ or internet$ or media$ or online$ or website$ or whatsapp)).ti,ab. (11061)

22 ((parent$ or mother$ or maternal$ or mum$ or father$ or paternal$ or dad$ or carer$ or caregiver$) adj3 (course$ or drop$ or drop-in or group$ or home$ or program$ or represent$ or train$ or workshop$)).ti,ab. (60179)

23 18 or 19 or 20 or 21 or 22 (130702)

24 12 and 23 (952)

25 deaf cafe.ti,ab. (0)

26 (teacher$ adj3 deaf$).ti,ab. (175)

27 cued speech.ti,ab. (69)

28 (british sign language adj2 (class$ or lesson$)).ti,ab. (0)

29 (BSL adj2 (class$ or lesson$)).ti,ab. (13)

30 (sign language adj2 (class$ or lesson$)).ti,ab. (11)

31 (family sign language adj2 (class$ or lesson$)).ti,ab. (0)

32 deaf role model$.ti,ab. (4)

33 baby beat$.ti,ab. (0)

34 deaf instructor$.ti,ab. (2)

35 "sign and play".ti,ab. (18)

36 "sing and sign".ti,ab. (0)

37 marschak.ti,ab. (9)

38 theraplay.ti,ab. (19)

39 filial therapy.ti,ab. (17)

40 auditory verbal therapy.ti,ab. (46)

41 25 or 26 or 27 or 28 or 29 or 30 or 31 or 32 or 33 or 34 or 35 or 36 or 37 or 38 or 39 or 40 (378)

42 17 or 24 or 41 (3127)

43 limit 42 to embase (1389)

**MEDLINE**

**Via OVID**

**Search date=16/09/2019**

**Records retrieved = 2052**

**Database: Ovid MEDLINE(R) ALL <1946 to September 13, 2019>**

1 exp Child/ (1848005)

2 exp Infant/ (1106485)

3 (child$ or infant$ or infancy or preschool$ or pre school$ or baby or babies or pediat$ or paediat$).ti,ab. (1784852)

4 (boy or boys or girl or girls or youngster$).ti,ab. (221919)

5 1 or 2 or 3 or 4 (2973508)

6 Persons With Hearing Impairments/ (2515)

7 exp Hearing Disorders/ (84107)

8 exp Deaf-blind Disorders/ (1020)

9 6 or 7 or 8 (85087)

10 5 and 9 (27659)

11 ((child$ or infant$ or infancy or preschool$ or pre school$ or baby or babies or pediat$ or paediat$) adj4 (deaf$ or hearing$ or hard-of-hearing or listen$)).ti,ab. (13484)

12 10 or 11 (31481)

13 exp Parents/ (105921)

14 Parenting/ (15569)

15 exp Parent-Child Relations/ (54672)

16 exp Caregiver/ (33717)

17 13 or 14 or 15 or 16 (179684)

18 12 and 17 (1368)

19 ((parent$ or mother$ or maternal$ or mum$ or father$ or paternal$ or dad$ or carer$ or caregiver$) adj3 intervention$).ti,ab. (10304)

20 ((parent$ or mother$ or maternal$ or mum$ or father$ or paternal$ or dad$ or carer$ or caregiver$) adj3 (advice$ or advisory or counsel$ or helpline$ or information$ or pack$ or signpost$ or support$ or telephone$ or therap$ or visit$)).ti,ab. (39281)

21 ((parent$ or mother$ or maternal$ or mum$ or father$ or paternal$ or dad$ or carer$ or caregiver$) adj3 (advisor$ or audiolog$ or consultant$ or instructor$ or psychologist$ or teacher$ or technician$ or therapist$)).ti,ab. (8363)

22 ((parent$ or mother$ or maternal$ or mum$ or father$ or paternal$ or dad$ or carer$ or caregiver$) adj3 (email$ or facebook$ or internet$ or media$ or online$ or website$ or whatsapp)).ti,ab. (7076)

23 ((parent$ or mother$ or maternal$ or mum$ or father$ or paternal$ or dad$ or carer$ or caregiver$) adj3 (course$ or drop$ or drop-in or group$ or home$ or program$ or represent$ or train$ or workshop$)).ti,ab. (42492)

24 19 or 20 or 21 or 22 or 23 (94413)

25 12 and 24 (691)

26 deaf cafe.ti,ab. (0)

27 (teacher$ adj3 deaf$).ti,ab. (171)

28 cued speech.ti,ab. (61)

29 (british sign language adj2 (class$ or lesson$)).ti,ab. (0)

30 (BSL adj2 (class$ or lesson$)).ti,ab. (10)

31 (sign language adj2 (class$ or lesson$)).ti,ab. (12)

32 (family sign language adj2 (class$ or lesson$)).ti,ab. (0)

33 deaf role model$.ti,ab. (4)

34 baby beat$.ti,ab. (1)

35 deaf instructor$.ti,ab. (2)

36 "sign and play".ti,ab. (14)

37 "sing and sign".ti,ab. (0)

38 marschak.ti,ab. (12)

39 theraplay.ti,ab. (5)

40 filial therapy.ti,ab. (15)

41 auditory verbal therapy.ti,ab. (31)

42 26 or 27 or 28 or 29 or 30 or 31 or 32 or 33 or 34 or 35 or 36 or 37 or 38 or 39 or 40 or 41 (336)

43 18 or 25 or 42 (2052)

**PsycINFO**

**Via OVID**

**Search date=16^th^ September 2019**

**Records retrieved=2469**

**Database: PsycINFO <1806 to September Week 2 2019>**

1 (childhood birth 12 yrs or infancy 2 23 mo or neonatal birth 1 mo or preschool age 2 5 yrs).ag. (523760)

2 (child$ or infant$ or infancy or preschool$ or pre school$ or baby or babies or pediat$ or paediat$).ti,ab,id. (752126)

3 (boy or boys or girl or girls or youngster$).ti,ab,id. (103007)

4 1 or 2 or 3 (912570)

5 exp Deaf/ or exp Partially Hearing Impaired/ (13222)

6 exp Deaf Blind/ (280)

7 exp Hearing Disorders/ (19759)

8 5 or 6 or 7 (19759)

9 4 and 8 (9144)

10 ((child$ or infant$ or infancy or preschool$ or pre school$ or baby or babies or pediat$ or paediat$) adj4 (deaf$ or hearing$ or hard-of-hearing or listening-impair$)).ti,ab. (8405)

11 9 or 10 (11170)

12 exp Parents/ (112657)

13 exp Parenting/ (106401)

14 exp Parent Child Relations/ (66439)

15 exp Caregivers/ (26907)

16 12 or 13 or 14 or 15 (192454)

17 11 and 16 (1188)

18 ((parent$ or mother$ or maternal$ or mum$ or father$ or paternal$ or dad$ or carer$ or caregiver$) adj3 intervention$).ti,ab. (9595)

19 ((parent$ or mother$ or maternal$ or mum$ or father$ or paternal$ or dad$ or carer$ or caregiver$) adj3 (advice$ or advisory or counsel$ or helpline$ or information$ or pack$ or signpost$ or support$ or telephone$ or therap$ or visit$)).ti,ab. (33470)

20 ((parent$ or mother$ or maternal$ or mum$ or father$ or paternal$ or dad$ or carer$ or caregiver$) adj3 (advisor$ or audiolog$ or consultant$ or instructor$ or psychologist$ or teacher$ or technician$ or therapist$)).ti,ab. (22832)

21 ((parent$ or mother$ or maternal$ or mum$ or father$ or paternal$ or dad$ or carer$ or caregiver$) adj3 (email$ or facebook$ or internet$ or media$ or online$ or website$ or whatsapp)).ti,ab. (6067)

22 ((parent$ or mother$ or maternal$ or mum$ or father$ or paternal$ or dad$ or carer$ or caregiver$) adj3 (course$ or drop$ or drop-in or group$ or home$ or program$ or represent$ or train$ or workshop$)).ti,ab. (37736)

23 18 or 19 or 20 or 21 or 22 (91051)

24 11 and 23 (709)

25 17 or 24 (1545)

26 deaf cafe.ti,ab. (0)

27 (teacher$ adj3 deaf$).ti,ab. (393)

28 cued speech.ti,ab. (113)

29 (british sign language adj2 (class$ or lesson$)).ti,ab. (1)

30 (BSL adj2 (class$ or lesson$)).ti,ab. (3)

31 (sign language adj2 (class$ or lesson$)).ti,ab. (26)

32 (family sign language adj2 (class$ or lesson$)).ti,ab. (0)

33 deaf role model$.ti,ab. (8)

34 baby beat$.ti,ab. (0)

35 deaf instructor$.ti,ab. (4)

36 "sign and play".ti,ab. (3)

37 "sing and sign".ti,ab. (0)

38 marschak.ti,ab. (72)

39 theraplay.ti,ab. (112)

40 filial therapy.ti,ab. (229)

41 auditory verbal therapy.ti,ab. (50)

42 26 or 27 or 28 or 29 or 30 or 31 or 32 or 33 or 34 or 35 or 36 or 37 or 38 or 39 or 40 or 41 (990)

43 25 or 42 (2469)

**Science Citation Index**

**Via Web of Science**

**Search date=18^th^ September 2019**

**Records retrieved=406**


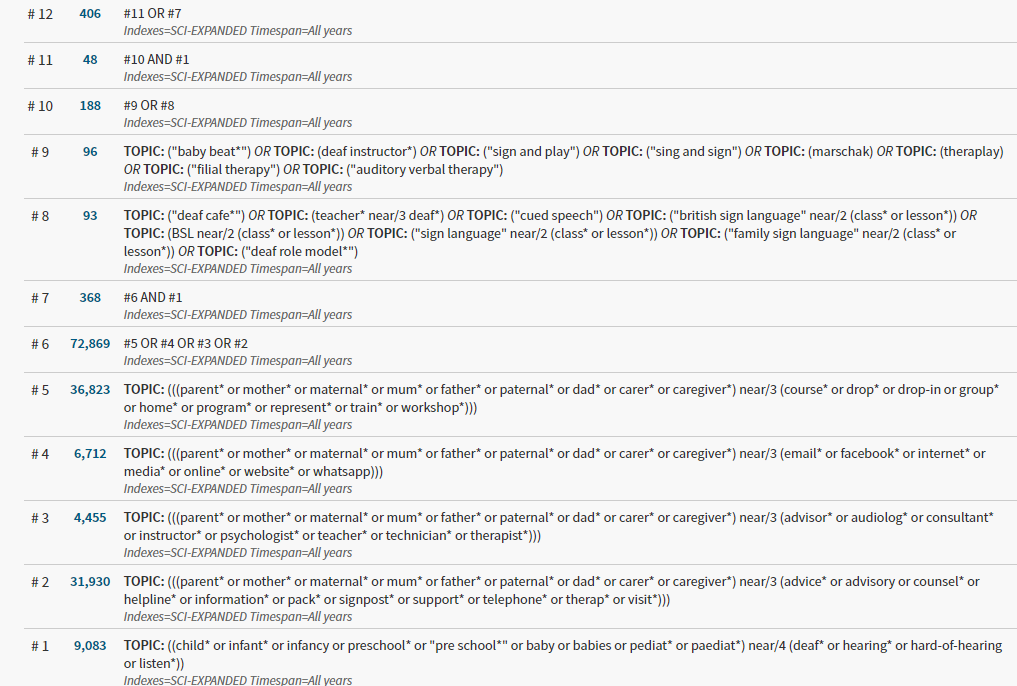


**Scopus**

**Search date=20^th^ September 2019**

**Two search strategies were carried out and the results were loaded into EndNore bibliographic software and deduplicated to leave 1390 records in total**

1137 records identified

( TITLE-ABS-KEY ( ( child* OR infant* OR infancy OR preschool* OR "pre school*" OR baby OR babies OR pediat* OR paediat* ) W/4 ( deaf* OR hearing* OR hard-of-hearing OR listen* ) ) ) AND ( ( TITLE-ABS-KEY ( ( parent* OR mother* OR maternal* OR mum* OR father* OR paternal* OR dad* OR carer* OR caregiver* ) W/3 ( advice* OR advisory OR counsel* OR helpline* OR information* OR pack* OR signpost* OR support* OR telephone* OR therap* OR visit* ) ) ) OR ( TITLE-ABS-KEY ( ( parent* OR mother* OR maternal* OR mum* OR father* OR paternal* OR dad* OR carer* OR caregiver* ) W/3 ( advisor* OR audiolog* OR consultant* OR instructor* OR psychologist* OR teacher* OR technician* OR therapist* ) ) ) OR ( TITLE-ABS-KEY ( ( parent* OR mother* OR maternal* OR mum* OR father* OR paternal* OR dad* OR carer* OR caregiver* ) W/3 ( email* OR facebook* OR internet* OR media* OR online* OR website* OR whatsapp ) ) ) OR ( TITLE-ABS-KEY ( ( parent* OR mother* OR maternal* OR mum* OR father* OR paternal* OR dad* OR carer* OR caregiver* ) W/3 ( course* OR drop* OR drop-in OR group* OR home* OR program* OR represent* OR train* OR workshop* ) ) ) )

313 records identified

( TITLE-ABS-KEY ( ( child* OR infant* OR infancy OR preschool* OR "pre school*" OR baby OR babies OR pediat* OR paediat* ) W/4 ( deaf* OR hearing* OR hard-of-hearing OR listen* ) ) ) AND ( ( TITLE-ABS-KEY ( {deaf cafe} OR {cued speech} OR {deaf role model} OR {deaf instructor} OR marschak OR theraplay ) ) OR ( TITLE-ABS-KEY ( {filial therapy} OR {auditory verbal therapy} OR {baby beats} OR {sign and play} OR {sing and sign} ) ) OR ( TITLE-ABS-KEY ( "sign language" W/2 class* ) ) OR ( TITLE-ABS-KEY ( "sign language" W/2 lesson* ) ) OR ( TITLE-ABS-KEY ( bsl W/2 ( lesson* OR class* ) ) ) OR ( TITLE-ABS-KEY ( teacher* W/3 deaf* ) ) )

**Social Science Citation Index**

**Via Web of Science**

**Search date 18^th^ September 2019**

**Records retrieved=574**


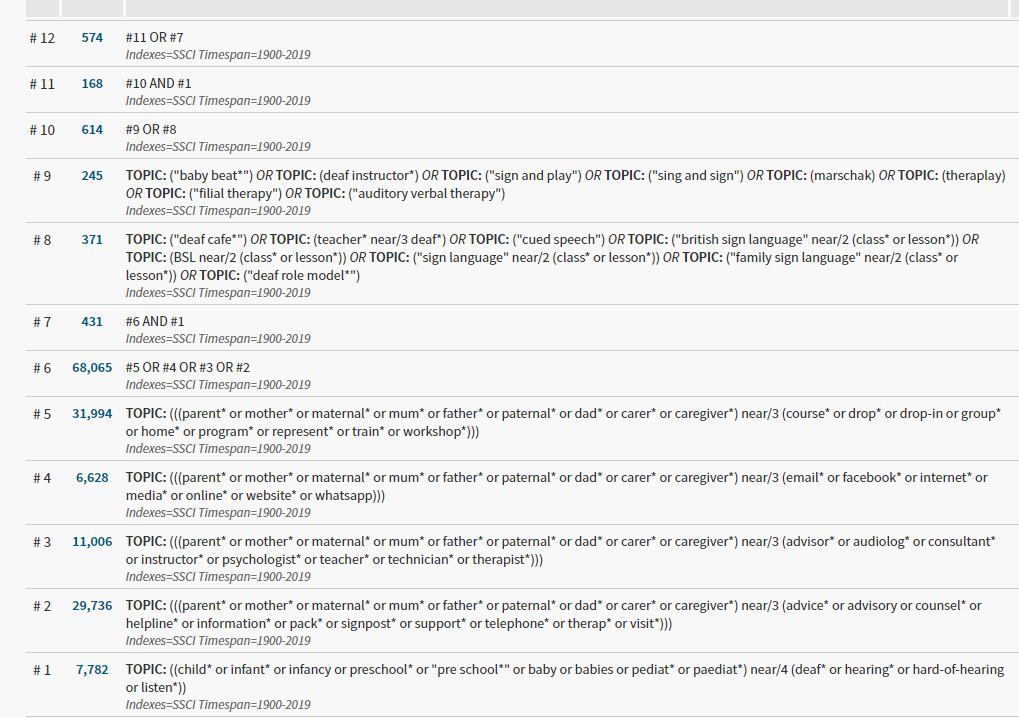

Supplement: Supplementary file 1 — Additional file 1. Full search strategy. [file 12887_2021_2893_MOESM1_ESM.docx]
