# Supplementary material for: A systematic scoping review of early interventions for parents of deaf infants
Source: BMC Pediatr. 2021 Oct 22;21:467. doi: 10.1186/s12887-021-02893-9 (PMC8532316; doi:10.1186/s12887-021-02893-9)
Supplement: Supplementary file 2 — Additional file 2: Table 1: Characteristics of Included Studies. [file 12887_2021_2893_MOESM2_ESM.docx]

**Table 1: Characteristics of Included Studies**

| **Author, Year** | **Study Design** | **Theme** | **Intervention** | **Rationale/**  **Theory** | **Delivery** | **Results** | **Risk of Bias** |
| --- | --- | --- | --- | --- | --- | --- | --- |
| Roberts, 2019 | RCT | Language and Communication | Parent-Implemented Communication Treatment (PICT) | Includes strategies (visual, interactive, tactile and linguistically stimulating) to promote early communication and parent sensitivity in everyday routine | Face to face, home sessions delivered by a deaf educator or a speech-language pathologist. Hour long sessions once per week over 6 months | There was a difference in parent use of treatment strategies between intervention and control group (effect size 1.08 (p = .04). There was a difference in gains in pre-linguistic speech skills between child intervention and control groups (effect size 1.09 (p = .03) | Some concerns |
| Glanemann, Reichmuth, Matulat, and am Zehnhoff-Dinnesen, 2013 | RCT | Language and Communication | Muenster Parenting Programme | Enhancing parents’ responsive communicative behaviour and reducing inappropriate communicative initiative behaviour | Six group sessions, two single training sessions with video feedback and two individual counselling sessions over 3 months. A group consists of 4–6 families (without children) held in a clinic | Trained parents enhanced responsiveness to child’s vocal, non-verbal and preverbal signals, and reduced inappropriate initiative behaviour. Significant increases in vocalisations for children in the training group | High Risk |
| Prinz and Masin, 1985 | Comparison Group - Matched Control | Language and Communication | Adult "Recasting" in sign language | In "recast" replies in conversation, the child's utterance is redisplayed in an altered sentence structure that still refers to the central meanings of the first sentence | Sessions took place in the child's home and school with four 30-minute sessions weekly (2 with mother/2 with teacher) for a total of 80 sessions. | Mothers and teachers significantly increased sign production.   Children demonstrated a significant increase over time in the number of sign utterances produced | Low |
| McConnell, 1974 | Comparison Group - Comparative Evaluation | Language and Communication | Bill Wilkerson Hearing and Speech Centre Program  1. Mama Lere Parent Teaching Home for parents of children 0-3 years 2. Acoustic preschool classes for children 3-6 years | To give parents of hearing impaired children immediate and continuing help in developing their child’s ability to understand and develop spoken language in the period before formal education | Delivered at a model home at the centre by a Teacher / Audiologist | Statistically significant differences were found between the late intervention group and both the early intervention and hearing groups. Children with early intervention performed at a similar level to the hearing groups | Moderate |
| Suskind, Graf, Leffel, Hernandez, Suskind, Webber, Tannenbaum, and Nevins, 2016 | Comparison Group – Quasi-experimental | Language and Communication | ASPIRE Intervention Curriculum | Caregiver-directed educational intervention curriculum designed to improve deaf children’s early language environments, based on using hearing devices and building vocabulary | Delivered at medical centres, community centres and through home visits by a certified early interventionist | Significant increases in caregiver knowledge questionnaire scores and language behaviour in the treatment but not the control group.   No statistically significant results for the children | Moderate |
| Hogan, Stokes and Weller, 2010 | Comparison Group | Language and Communication | Auditory Verbal (AV) Therapy | The primary aim is to develop listening behaviours in children who use hearing technologies.  AVT was provided to low income families, to tackle the misconception that families in AVT are mainly middle-class, affluent and well-educated | 1-hour therapy sessions on a fortnightly basis. The families were seen by up to 4 certified AV therapists during their programme of intervention | No significant difference between the Rate of Language Development (RLD) for the low income group children and the control group, both showed an accelerated RLD compared to the mean for typically hearing children | Moderate |
| Greenberg, Calderon, and Kusché, 1984 | Comparison Group - Matched Control | Language and Communication | The Counselling and Home Training Program for Deaf Children | The program aims to encourage rich and natural communication between deaf children and their families through counselling and supportive contact, and builds the deaf children's sense of competence and esteem by developing secure family context | 3 weekly visits, at home and at CHTP centre. One off events and activities, consultation sessions >1Y. Included an MDT approach | More developmentally mature communication and high interaction in intervention families, with children demonstrating a significantly higher number of questions and spontaneous communication than comparison children | Moderate |
| Ivey and Teel, 1974 | Comparison Group - Matched Control | Language and Communication | Tri-sensory Language Stimulation | Designed to enhance the sensory feedback of speech | 16 sessions (2 per week for 8 weeks) | A range of language measures representing 19 separate variables were used, nine of these variables had a significant effect | Not enough information |
| Venon and Koh, 1971 | Comparison Group - Matched Control (Longitudinal Follow Up) | Language and Communication | Tracy Clinic Oral Preschool | Focus on oral education | Not Reported | No significant differences in speech in the three groups. Deaf children of deaf parents scored higher on academic achievement tests compared to children in the intervention group with hearing parents and matched control deaf children with hearing parents | Moderate |
| DeSalle Howse and Fitch, 1972 | Comparison Group - Matched Control | Language and Communication | Parent Orientation Program | Early exposure to manual communication | Not Reported | Intervention group children showed larger increases in communication from minimal cues, number of signs and total communication attempts, and a smaller loss of natural gestures than the control group children | Moderate |
| Watkins, 1987 | Comparison Group - Longitudinal multiple comparison procedures | Language and Communication | The SKI*HI Program | The SKI*HI Program focuses on parents, who are taught how to provide auditory and language stimulation for the child in the home | Weekly home visits approximately 1-1.5 hours long with a SKI-HI parent advisor | Hearing-impaired children in this study who had received earlier home intervention performed better than children who had received no home intervention on the majority of language, academic achievement and social functioning variables | Moderate |
| Harrigan and Nikolopoulos, 2002 | Pre-Post prospective study | Language and Communication | Training course for parents designed to help them to be less controlling in their interactions | Encouraging parents to become more responsive partners in communication. Emphasis is placed on adapting spoken language and parents are taught communication facilitation | Each course consisted of three training days interspersed with home visits with Hanen certified speech and language therapists | Statistically significant reduction in post-course parent initiation and statistically significant increase in responses. 12 months later, the response score remained high | Low |
| Sacks, Shay, Repplinger, Leffel, Sapolich, Suskind, Tennenbaum and Suskind, 2014 | Pre-Post | Language and Communication | Project ASPIRE Education, quantitative linguistic feedback | To provide parents with critical knowledge and skills to help their children with hearing loss reach their fullest linguistic potential | One hour intervention home visit, parents completed three post-intervention recordings. Parents continued to receive quantitative linguistic feedback via phone sessions after each follow-up recording with a Deaf Educator | Post intervention increases in adult word count and significant post intervention increases in child vocalisation count and child-parent interactive behaviour | Low |
| Paganga, Tucker, Harrigan and Lutman, 2001 | Pre-Post | Language and Communication | Modified Hanen Program | The aim is to help parents gain information about language development and adopt interactive styles that promote their own child’s communication | Nine group teaching sessions and four individual sessions are provided for each parent with a Hanen Trained Therapist | Adult conversational fluency rating scores, pooled across raters and subjects, were significantly higher post intervention | Low |
| Ling, 1971 | Pre-Post | Language and Communication | Parent Counselling Program | Training was largely parent guidance in the use of hearing aids and the development of speech communication skills. Stress was placed on the use of hearing as the dominant modality in the development of speech and language skills | Weekly sessions of approximately 1 hour, on an individual basis for parents (mother and child generally) in a community setting (a pleasant but distraction free room) with a Therapist | Significant modification was achieved in several areas of ability assessed with the Griffiths Mental Development Scale, though deficits in personal-social adjustment and hearing and speech skills remained | Low |
| Dee, Rapin and Ruben, 1982 | Pre-Post | Language and Communication | Total Communication | Parents trained in total communication | Family instruction sessions (including parents, child and siblings) at school and home visits as required, weekly 2 hr sessions for 6-20 months with Teachers | Sign language facilitated the child's acquisition of communicative oral speech. Young children receiving total communication appeared to learn and express more language at an earlier age than is typical of orally trained hearing-impaired children | Low |
| Luetke-Stahlman and Moeller, 1990 | Pre-Post | Language and Communication | SSE-2 Support | SSE2 Intervention strategies designed specifically to meet the communication needs of hearing parents | Discuss signing ability and set out goals, videotaping interactions in play | SEE-2 consistency averaged 57% at baseline, 80% during intervention, and 67% during the retention taping. Overall, the group gained an average consistency rating of 13% across all phases of the study | Low |
| Lund, 2018 | Pre-Post (Multiple Baseline) | Language and Communication | Parent training focused on transparent labelling and linguistic mapping strategies | Parent-training intervention centred on two language facilitation strategies: transparent labelling and linguistic mapping | Both children and parents attended a 1 hour parent-training session at the clinic, weekly for 4 weeks. Forty-five minutes of therapy (according to condition), and 15 minutes of probe assessments | A relationship between parent training and use of transparent labelling was established for all mothers. Child vocabulary growth rate increased from baseline to intervention in 4 of 6 children | Moderate |
| Hogan, Stokes, White, Tyszkiewicz and Woolgar, 2008 | Other - Predicted language scores in the absence of intervention were calculated according to a model | Language and Communication | Auditory - Verbal Therapy | The Auditory-Verbal approach puts parents and carers at the centre of the intervention programme. They receive training and knowledge through therapy sessions, practising techniques, and developing strategies for integrating them into daily living | 60 or 90 minutes - twice a month for a minimum of 12 months with Certified AV therapists | For all age groups and for each of the different hearing technologies, AVT was found to be a highly effective programme for accelerating spoken language development measured by rate of language development (RLD). There was no significant difference in RLD between children aided before or after 6 months | N/A |
| Calderon and Low, 1998 | Other - Sub analysis of larger study looking at presence of fathers | Language and Communication | Early Childhood Home Instruction (ECHI) | The program emphasises language and communication development using auditory and speech training and manual communication | Parent support group and centre based play group to promote language development. 1-2 hour visits averaging 2.9 sessions per month for 15 months | Results indicate that children with a father present demonstrated stronger language skills immediately following early intervention and, more important, that those differences held across time compared to results for those children without a father present | N/A |
| Suzuki and Notoya, 1984 | Other - Longitudinal | Language and Communication | Language Training | Based on the theory that learning of written language will facilitate the learning of oral language | A two hour group session for mothers once every two weeks with Teachers | Acquired vocabulary in written language was significantly greater than that of oral language at age 23 months, at 35 months, at 47 months, and at 59 months | N/A |
| Simmons-Martin, 1981 | Other - Longitudinal | Language and Communication | Central Institute for the Deaf Early Education Project | Encourages parent-child verbal interaction through cognitively challenging activities and parental guidance in emotional relationship with child | Not Reported | Scales of Early Communication Skills for hearing impaired children examined every 6 months for 2.5 years - trend analysis performed for mean scale values over each age span found reliable linear increase for all ages | N/A |
| Takala, Kuusela and Takala, 2000 | Other - 5-year longitudinal | Language and Communication | A good future for deaf children | 5 year educational project teaching sign language | Child and deaf teacher have a 2-4 hour meeting, weekly for 1 year. Families study sign language. All project families signed together twice yearly and teaching was delivered to day care staff | The families indicated satisfaction with the project; they learned to sign and their social networks expanded. Parents favoured bilingual education. The families that were most actively involved in the lessons learnt the most | N/A |
| Easterbrooks, O'Rourke and Todd, 2000 | Other – Retro-spective case review | Language and Communication | Auditory–Verbal Therapy | Intervention approach that focuses on early identification and amplification for children with hearing loss | Delivered by a therapist at a clinic - duration varied but with a minimum of 1 year | Those children who stayed in the program until graduation were less likely to have an educationally significant communication gap and had a high probability of being completely included in regular education with no support from a deaf educator | N/A |
| Thompson and Swisher, 1985 | Other - Randomly selected file review | Language and Communication | The 10-year-old Early Childhood Home Instruction Program for Hearing-Impaired Infants and Their Families | Total communication including various modes of communication such as speech, speech-reading and auditory training (using amplification), supported by fingerspelling and sign language) | 10 - 24 month duration | Results showed a gain in receptive language skills of 22.86 months, and of expressive language skills of 21.62 months. Seven of the 13 children learned to use speech spontaneously. Group language gains were seen even though not all parents followed through on all aspects of the program | N/A |
| Daczewitz, 2015 | Other - Single case study multiple baseline design | Language and Communication | PiCS intervention using distance education technology (naturalistic teaching strategies for communication) | Parents were trained and coached to use naturalistic teaching strategies (i.e., environmental arrangement, modelling, mand-model, and time delay) | Online coaching via skype. Baseline phase sessions 1-2 times per week and Coaching phase sessions include modelling, coaching, setting goals and completing observations. Maintenance phase used observations only | Increase in high quality use of strategies with a combined analysis for all three targeted strategies showing an overall effect size of 0.6970. Child's performance on the Communication Developmental Inventories and Cottage Acquisition Scales improved from baseline to post-intervention | N/A |
| Steinberg, 1982 | Other - Case Study | Language and Communication | A program for teaching written language | A four phase teaching program was formulated incorporating; word familiarisation, word identification, phrase and sentence identification and text interpretation | Home based - 10-30 mins per day for 8-20 months. Teachers taught parents to complete the programme | An increase in the number of words and phrases known by child and significant written language knowledge was gained | N/A |
| Gaines and Halpern-Felsher, 1995 | Other - Observational Case Study | Language and Communication | HI CHIPS Total Communication Program | Total communication program. Infants were taught sign and vocal language. Parents were tutored in simultaneous English sign and vocal language | Group meets 3 mornings a week for 3 hour sessions with a specially trained Teacher of the Deaf | Comparisons between the deaf and hearing twins showed that both children were able to learn language and communicate successfully. No significant differences were found between twins in production of spontaneous signs | N/A |
| Seitz and Marcus, 1976 | Other - Case History | Language and Communication | Communication Program | Not reported | Each mother spent 2 weeks observing and discussing therapist-child interactions prior to observed interactions for short periods with feedback. 1 hour sessions, 4 times a week for 20 weeks. | There was an increase in positive responding between parent and child, accompanied by a reduction in the mother's directive behaviour. For children there was an absence of negative and unresponsive behaviour and an increase in number of utterances | N/A |
| Costa, Day, Caverly, Mellon, Ouellette and Wilson Ottley, 2019 | Comparison Group - Matched Control (subset analysis) | Parent Knowledge and Skills | Parent–Child Interaction Therapy (PCIT) | PCIT actively teaches parents strategies to apply at home and improves maternal sensitivity, parental responsiveness, and emotional availability | School based clinic - Average length of treatment for all in matched experimental group was 15.6 sessions (5.8 CDI, 7.7 PDI). Delivered by a Clinical psychologist who is certified by PCIT International | Significant changes in appropriate parent language use from pre to post treatment in the PCIT group. A subset of the treatment group showed a significant increase in utterances compared to the matched control. Also a  significant improvement in child behaviour | Moderate |
| Dirks and Wauters, 2018 | Comparison Group | Parent Knowledge and Skills | Interactive storybook reading | Interactive reading may be particularly beneficial for deaf children, but research has shown a lack of reading training programs for hearing parents of deaf children | Three 2-h group sessions over a period of 6 weeks with 2 professionals (an early interventionist and a speech and language therapist), followed a one-day training from the program developers and a manual | The gain scores were significantly larger in the experimental group than in the comparison group for total parent behaviour, engagement teacher techniques, and interactive reading. No differences in improvement were found for literacy strategies | Moderate |
| Watkins, Pittman, Walden, 1998 | Comparison Group - Matched Control | Parent Knowledge and Skills | Utah Deaf Mentorship Programme | The mentor focused primarily on three areas: (a) teaching the family ASL, (b) interacting with the child using ASL, and (c) teaching the family about Deaf culture and introducing the family to the local Deaf community | 1-2 hours parent advisor sessions as weekly home visits with National SKI-HI trainers  Average amount of deaf mentor home visiting per month = 6.5 hours, average number of mentor visits per month = 4 times per month - 17.6 months of deaf mentor treatment | Parents in the intervention group used more than six times as many signs as the control parents and became more comfortable using ASL and signed English. Intervention group children demonstrated greater gains in receptive and expressive language during treatment and scored higher at post-test on grammatical structures than the control group | Not enough information |
| Perry, 1986 | Comparison Group - Control Group | Parent Knowledge and Skills | Parent Study Group | The Adlerian Model stresses expectations and encouragement | 8 group sessions, delivered by the researcher | No statistically significant differences were found between the treatment group and the control group although the treatment group did improve along more dimensions (Adlerian Parental Assessment of Child Behaviour Scale (APACBS) than the control group | Moderate |
| Nelson, Stoddard, Fryer, and Muñoz, 2019 | Pre-Post (Multiple case pilot study) | Parent Knowledge and Skills | Training session focused on storybook reading | Parent–child shared reading can be optimized when literacy engagement strategies are directly taught to parents | Each participant engaged in a 20-min training session in a quiet room at the child’s preschool classroom. Parents then continued with their 15-min book reading over a 2-week period to obtain four to five recorded sessions. Study authors completed the training for all families | After training, parent participants increased language expansion and critical thinking opportunities for their child by utilising engagement prompts.  Average child contributions to the reading interactions increased from 14% at baseline to 22% after intervention | Low |
| Delk and Weidekamp, 2001 | Other - Logic Model Evaluation | Parent Knowledge and Skills | The Shared Reading Project | The Shared Reading Project is built on the premise that hearing people can learn to read storybooks to deaf and hard of hearing children by observing how deaf adults do it | A trained Shared Reading tutor (from a range of backgrounds and professions) visits the family home once a week with a specially designed book bag. Each week for 20 weeks, the tutor brings a new bag to the family | During the Shared Reading Project, participating families shared books an average of 5.2 times a week, compared to figures from the general population which suggests that 83% of 3-to 5-year-old children were read to three or more times a week by a family member | N/A |
| Mueller, 2008 | Other - Case Study | Parent Knowledge and Skills | Iowa E-Book | The books are designed to assist and simulate the lap-reading experiences deemed so predictive of later academic success in hearing children | 5 weeks; baseline, treatment 1, withdrawal (no sign support), treatment 2, withdrawal, wrap up. Delivered by a researcher.  Video first 5 minutes of each session | Highly mixed results reported.  For the children, the same proportion of signs was learnt in both sign and non-sign support books. They learned a greater proportion of vocabulary during non-signed phases, but spent longer reading during sign support | N/A |
| Foust and Wynne, 1991 | Other -Experimental Design (single subject, alternating treatments design) | Parent Knowledge and Skills | Parent training program on performing adequate listening checks on hearing aids | The audiologist traditionally provides the initial hearing aid fitting, orientation, and follow-up. Due to this, actual parent or educator training in amplification and their practice with hearing aids is often limited or non-existent | A total of six treatment sessions over a two week period in the clinic with a clinician | A large increase in frequency of target behaviours (required to perform adequate listening and visual inspection of a hearing aid) for parents receiving clinician directed treatment | N/A |
| Janssen, Riksen-Walraven, and Van Dijk, 2003 | Other - Series of Case Studies (2 fit inclusion criteria) | Parent Knowledge and Skills | Educator-oriented contact intervention program | The interaction training framework is inspired by various models and approaches, including the model developed by the DbI European Working Group on Communication, the model used by Nafstad and Rodbroe (1999), and the principles of video home training | The number of coaching sessions depended on the expertise of the educator and the complexity of the target behaviours and ranged from 0-10 sessions. Delivered by Interaction Coaches. | For one child, positive intervention effects were observed for all the target behavioural categories with the exception of approving answers of the educator. For the second child, positive intervention effects were observed for all the target categories, with the exception of confirmation by the educator | N/A |
| Garrard and Saxon, 1973 | Other - Case Study | Parent Knowledge and Skills | Treatment program | Much of the treatment for the mothers benefit - to make her feel on a par with professionals | Read information and meetings to discuss. Meetings at support/clinic and home. Up to 34 sessions (2 per week). Delivered by a Psychologist and speech pathologist. | Reduction in adverse child behaviour and an increase in child attending/ approaching during tasks | N/A |
| Bergeron, 2013 | Other - Single case Design | Parent Knowledge and Skills | Parent Training on Storybook Reading | Part of a larger study testing 'Foundations for Literacy' preschool literacy curriculum, parenting training which targets 3 strategies through explicit instruction and support for storybook reading with child | Recording a shared book reading experience at least 4 times a week for 12 weeks and attending 3 parent training workshops every 4 weeks | No effects found.  Results showed this was not effective as an intervention to change parent behaviour during shared reading | N/A |
| Dromi and Ingber, 1999 | Other - Qualitative | Parent Knowledge and Skills | Family-oriented early intervention program | Provision of information, guidance, and support to empower parents to build a collaborative partnership in order to develop competence and involvement in their child’s education and development | Not enough information provided | The mothers generally sought information, guidance, and support from the intervention and hoped that professionals would be well trained and proficient in interpersonal skills, high- lighting cognitive, behavioural, and emotional issues | N/A |
| Snoddon, 2009 | Other Qualitative | Parent Knowledge and Skills | ASL Parent - Child Mother Goose programme | The program aims to strengthen the bond between mother and child through developing parental skills | Group sessions held at a Deaf Service Agency. 8 week delivered by an ASL Parent-Child Mother Goose Program Coordinator and senior ASL, literacy consultant and university ASL instructor | The study found benefits of accessing ASL as a native signed language for young deaf children in terms of cognitive, affective and academic development. This should therefore be supported by governmental programs and services | N/A |
| Mostafavi, Oryadi-Zanjani, Rad, Hazavehei, Rezaianzadeh and Ravanyar, 2017 | RCT | Parental Wellbeing and Empowerment | Educational intervention program based on empowerment of mothers | A training package including a protocol - includes initial evaluation, mothers' awareness and knowledge regarding the necessary interventions in hearing-impaired children, parenting, resilience, increased self- confidence, and parental empowerment | Family based face to face intervention held at a Speech and Language Centre. 9, 80 minute sessions held at regular intervals. Sessions held with the participation of researchers, a speech therapist, and a psychologist. | There was a significant difference for mothers' empowerment and self-efficacy between pre-test and follow-up. For children, there was a reduction in the severity of speech disorders between pre-test, post-test and follow-up | Some concerns |
| Adams and Tidwell, 1989 | RCT | Parental Wellbeing and Empowerment | Self-instructional parenting program | Coping and behaviour management program designed specifically for parents. Addresses feelings, attitudes and perceptions of interactions and teaches behaviour management | Eight week self-instructional parent program completed at home. Investigators led the program of self-directed intervention. | No significant differences between groups were found on any of the measures (Parenting Stress Index, Questionnaire on Resources on Stress, Child Behaviour Scale, Eyberg Child Behaviour Inventory) | Some concerns |
| Aiello and Ferrari, 2015 | RCT | Parental Wellbeing and Empowerment | Babies Portal social network | An online social network aims at connecting individuals, providing social relationships, gathering users with similar interests, content, location, learning, and mutual aid | Online access. Two Speech Language Pathologists and a Psychologist, acted as moderators in the social network, by proposing discussion topics and answering questions | No difference was observed in mean Parental Stress Index scores between the groups, except for the “Defensive Response” subscale, in which a decrease was observed in the control groups score | Some concerns |
| Greenberg, 1983 | Comparison Group - Matched Control | Parental Wellbeing and Empowerment | The Counselling and Home Training Program for Deaf Children | This family-oriented program aims to; improve natural communication by utilising all modes of communication, provide support through contact with other parents and deaf people and to develop a secure family context | 3 weekly visits, one off events and activities, consultation sessions, held at home and at CHTP centre with an MDT approach. | Mothers in the intervention group reported significantly lower overall stress compared to comparison mothers. Quality of attachment and overall behaviour adjustment were enhanced in children in the intervention group | Moderate |
| Kargin, 2004 | Comparison Group - Matched Control | Parental Wellbeing and Empowerment | Family-focused early intervention program | Focus on early intervention programs for parents in rural areas with children with disabilities and special needs. Program includes effective usage of a community's resources for child education, home based training, and improving attitudes of the community | Sessions at home. Information sessions pre intervention: 1 x 2-hour session in week 1 and 2 x 2-hour sessions in week 2. Intervention phase: 4 weeks of sessions 2x week, 3 weeks with sessions once a week. Delivered by the researcher | Experimental group scored significantly lower than the control on PND (parental needs determination scale) indicating less need for information. Post-test, the experimental group children scored significantly higher than the control on verbal communication skills | Moderate |
| Havenga, Swanepoel, Le Roux and Schmid, 2017 | Pre-Post (Within-subject design) | Parental Wellbeing and Empowerment | Tele-intervention sessions | Tele-intervention could be used to overcome barriers like the shortage of trained early-intervention providers and the high costs of providing services to geographically dispersed families | Tele-intervention sessions were conducted with the parent and child in their own home, communicating with the clinician in her office via Skype. Conventional intervention sessions were conducted by the clinician participant at the Centre | No significant differences between conventional and tele-intervention for parents in terms of facilitating meaningful communication interaction or for communication performance of children.   Conventional sessions were rated significantly higher on comfort level, being a beneficial experiences, and wanting to be continued in future | Moderate |
| Backenroth, 1984 | Pre-Post | Parental Wellbeing and Empowerment | Parent Group Counselling Programme | Group counselling, being primarily remedial in nature, support and empathy was stressed - reaction skills, interaction skills and action skills | The groups gathered every other week. The groups were closed after 15 sessions, which extended over a period of two semesters. Delivered by a counsellor. | Parents' interaction in the group showed significant differences before-and-after group counselling in 8 of 11 variables | Moderate |
| Störbeck and Pittman, 2008 | Pre-Post | Parental Wellbeing and Empowerment | The HI HOPES model of early intervention | The HI HOPES model of early intervention advocates a comprehensive, multidisciplinary approach to supporting families with the central aim of informing and equipping parents to make their own decisions based on the particular needs of their infant | Weekly lessons at home, 1 - 1.5 hours long. Parents work with a trained deaf mentor to learn about deaf culture and South African Sign Language.  Utilises an MDT approach (including parent advisors, deaf mentors, parents, HI HOPES team) | Parents reported high satisfaction with the program. 10 children showed an average overall language increase of 4.66 months per quarter, with the average receptive language increasing 4.23 months and the average expressive language increasing 5.1 months over the four- month cycles | Moderate |
| Moeller, 2000 | Other - Retrospective survey | Parental Wellbeing and Empowerment | Diagnostic Early Intervention Program (DEIP) | This multidisciplinary family-centred program is designed to support families of recently diagnosed children in identifying needs and making decisions related to intervention options | 1 - 2 home visits weekly, and a parent support group. Weekly family sign class available. The average duration of the family’s enrolment was 15 months | There was a significant negative correlation between age of enrolment and language outcomes at 5 years. Family involvement explained the most variance | N/A |
| Lam-Cassettari, Wadnerkar-Kamble and James, 2015 | Comparison Group - Stratified wait list control | Parent and Child Relationship | Family-focused psychosocial video intervention program | The intervention uses video feedback of spontaneous parent–child interactions to increase appropriate responsiveness to a child’s communicative cues, and promote attuned behaviours between parent and child | Consists of a goal setting session; 3 filming sessions of parent–child interaction in the family home, and three shared review sessions, which lasted 45 minutes. The film and shared review sessions were 2 separate visits, each took place once per month | The intervention group showed increases in scores on the Emotional Availability subscales, and in reported self-esteem | Low |
| Koohi, Sajedi, Movallali, Dann and Soltani, 2016 | Comparison Group - Quasi-experimental with pre-test-post-test and control group | Parent and Child Relationship | Faranak Parent-Child Program (Persian version of parent-child mother goose program) | The program aims to strengthen the bond between mother and child. The Mother Goose program is a group program in which parents participate with their children, and most activities are based on poetry, songs and storytelling | The Parent-Child Mother Goose Program was presented to the participants across 12 group sessions in which parents participate with their children | The total scoring of scales reveals an overall improvement in the mother- child relationship for those experiencing the intervention | Moderate |
| James, Waderkar-Kamble and Lam-Cassettari, 2013 | Pre-Post (Multiple pre- and post-intervention measures) | Parent and Child Relationship | Video Interaction Guidance (VIG) | Uses video recordings to highlight positive aspects of interaction and promotes these | Intervention delivery at home over 7 sessions. Includes a goal-setting session, 3 filming sessions and 3 review sessions  Training in the intervention approach is accredited in the UK by the Community of Practice of Associated Video Interaction Guidance Practitioners | Results for each case showed improvements in the sensitivity and structuring subscales on the Emotional Availability (EA) measure after the intervention and maintained at follow-up.   For children, the EA responsiveness and involvement subscales showed improvement. Tait Analysis showed trends for increased autonomy, and reduction of non-responses post intervention | Low |

**Systematic Review references**

Adams, J.W. and Tidwell, R., 1989. An instructional guide for reducing the stress of hearing parents of hearing-impaired children. American Annals of the Deaf, pp.323-328.

Aiello, C.P. and Ferrari, D.V., 2015. Teleaudiology: efficacy assessment of an online social network as a support tool for parents of children candidates for cochlear implant. Language, 15(9.3), pp.13-4.

Backenroth, G., 1984. Counselling in families with a deaf or hearing impaired child. International Journal for the Advancement of Counselling, 7(4), pp.267-274.

Bergeron, J.P., 2013. Effectiveness of Parent Training on Shared Reading Practices in Families with Children who are Deaf and Hard of Hearing.

Calderon, R. and Low, S., 1998. Early social-emotional, language, and academic development in children with hearing loss: Families with and without fathers. American Annals of the Deaf, pp.225-234.

Costa, E.A., Day, L., Caverly, C., Mellon, N., Ouellette, M. and Wilson Ottley, S., 2019. Parent–Child Interaction Therapy as a Behavior and Spoken Language Intervention for Young Children With Hearing Loss. Language, Speech, and Hearing Services in Schools, 50(1), pp.34-52.

Daczewitz, M., 2015. Delivering The Parent-Implemented Communication Strategies (pics) Intervention Using Distance Training And Coaching With A Father And His Child Who Is Hard Of Hearing.

Dee, A., Rapin, I. and Ruben, R.J., 1982. Speech and language development in a parent-infant total communication program. The Annals of otology, rhinology & laryngology. Supplement, 97, pp.62-72.

Delk, L. and Weidekamp, L., 2001. Shared Reading Project: Evaluating Implementation Processes and Family Outcomes. Sharing Results. National Deaf Education Network and Clearinghouse Product Inquiries, KDES PAS-6, 800 Florida Avenue, NE, Washington, DC 20002-3695.

DeSalle Howse, J.M. and Fitch, J.L., 1972. Effects of parent orientation in sign language on communication skills of preschool children. American annals of the deaf, pp.459-462.

Dirks, E. and Wauters, L., 2018. It takes two to read: Interactive reading with young deaf and hard-of-hearing children. The Journal of Deaf Studies and Deaf Education, 23(3), pp.261-270.

Dromi, E. and Ingber, S., 1999. Israeli mothers' expectations from early intervention with their preschool deaf children. Journal of Deaf Studies and Deaf Education, 4(1), pp.50-68.

Easterbrooks, S.R., O'Rourke, C.M. and Todd, N.W., 2000. Child and Family Factors Associated With Deaf Children's Success in Auditory–Verbal Therapy. Otology & Neurotology, 21(3), pp.341-344.

Foust, T.E. and Wynne, M.K., 1991. Effectiveness of supplemental parent training in hearing aid checks. Journal of the Academy of Rehabilitative Audiology.

Gaines, R. and Halpern-Felsher, B.L., 1995. Language preference and communication development of a hearing and deaf twin pair. American annals of the deaf, pp.47-55.

Garrard, K.R. and Saxon, S.A., 1973. Preparation of a disturbed deaf child for therapy: A case description in behavior shaping. Journal of Speech and Hearing Disorders, 38(4), pp.502-509.

Glanemann, R., Reichmuth, K., Matulat, P. and am Zehnhoff-Dinnesen, A., 2013. Muenster Parental Programme empowers parents in communicating with their infant with hearing loss. International journal of pediatric otorhinolaryngology, 77(12), pp.2023-2029.

Greenberg, M.T., 1983. Family stress and child competence: The effects of early intervention for families with deaf infants. American Annals of the Deaf, pp.407-417.

Greenberg, M.T., Calderon, R. and Kusché, C., 1984. Early intervention using simultaneous communication with deaf infants: The effect on communication development. Child Development, pp.607-616.

Harrigan, S. and Nikolopoulos, T.P., 2002. Parent interaction course in order to enhance communication skills between parents and children following pediatric cochlear implantation. International Journal of Pediatric Otorhinolaryngology, 66(2), pp.161-166.

Havenga, E., Swanepoel, D.W., Le Roux, T. and Schmid, B., 2017. Tele-intervention for children with hearing loss: A comparative pilot study. Journal of telemedicine and telecare, 23(1), pp.116-125.

Hogan, S., Stokes, J. and Weller, I., 2010. Language outcomes for children of low-income families enrolled in auditory verbal therapy. Deafness & Education International, 12(4), pp.204-216.

Hogan, S., Stokes, J., White, C., Tyszkiewicz, E. and Woolgar, A., 2008. An evaluation of auditory verbal therapy using the rate of early language development as an outcome measure. Deafness & education international, 10(3), pp.143-167.

Ivey, L.P. and Teel, J.R., 1974. Tri-Sensory Language Stimulation With The TAVF Unit. American annals of the deaf, pp.318-320.

James, D.M., Wadnerkar‐Kamble, M.B. and Lam‐Cassettari, C., 2013. Video feedback intervention: a case series in the context of childhood hearing impairment. International journal of language & communication disorders, 48(6), pp.666-678.

Janssen, M.J., Riksen-Walraven, J.M. and Van Dijk, J.P., 2003. Contact: Effects of an intervention program to foster harmonious interactions between deaf-blind children and their educators. Journal of Visual Impairment & Blindness, 97(4), pp.215-229.

Kargin*, T., 2004. Effectiveness of a family‐focused early intervention program in the education of children with hearing impairments living in rural areas. International Journal of Disability, Development and Education, 51(4), pp.401-418.

Koohi, R., Sajedi, F., Movallali, G., Dann, M. and Soltani, P., 2016. Faranak Parent-Child Mother Goose Program: Impact on Mother-Child Relationship for Mothers of Preschool Hearing Impaired Children. Iranian Rehabilitation Journal, 14(4), pp.201-210.

Lam-Cassettari, C., Wadnerkar-Kamble, M.B. and James, D.M., 2015. Enhancing parent–child communication and parental self-esteem with a video-feedback intervention: outcomes with prelingual deaf and hard-of-hearing children. Journal of deaf studies and deaf education, 20(3), pp.266-274.

Ling, A.H., 1971. Changes in the abilities of deaf infants with training. Journal of Communication Disorders, 3(4), pp.267-279.

Luetke-Stahlman, B. and Moeller, M.P., 1990. Enhancing parents' use of SEE-2: Progress and retention. American Annals of the Deaf, 135(5), pp.371-378.

Lund, E., 2018. The effects of parent training on vocabulary scores of young children with hearing loss. American Journal of Speech-Language Pathology, 27(2), pp.765-777.

Mcconnell, F., 1974. The parent teaching home: An early intervention program for hearing‐impaired children. Peabody Journal of Education, 51(3), pp.162-170.

Moeller, M. P. (2000). Early intervention and language development in children who are deaf and hard of hearing.

Mostafavi, F., Oryadi-Zanjani, M.M., Rad, G.S., Hazavehei, S.M.M., Rezaianzadeh, A. and Ravanyar, L., family-based training program: the role of mothers'empowerment in the speech development of children with hearing impairments.

Mueller, V.T., 2008. The effects of a fluent signing narrator in the Iowa E-Book on deaf children's acquisition of vocabulary, book related concepts, and enhancement of parent-child lap-reading interactions.

Nelson, L.H., Stoddard, S.M., Fryer, S.L. and Muñoz, K., 2019. Increasing Engagement of Children Who Are DHH During Parent–Child Storybook Reading. Communication Disorders Quarterly, 41(1), pp.12-21.

Paganga, S., Tucker, E., Harrigan, S. and Lutman, M., 2001. Evaluating training courses for parents of children with cochlear implants. International journal of language & communication disorders, 36(S1), pp.517-522.

Perry, D., 1986. PARENTING A HEARING IMPAIRED CHILD: AN ADLERIAN APPROACH (ARIZONA).

Prinz, P.M. and Masin, L., 1985. Lending a helping hand: Linguistic input and sign language acquisition in deaf children. Applied Psycholinguistics, 6(4), pp.357-370.

Sacks, C., Shay, S., Repplinger, L., Leffel, K.R., Sapolich, S.G., Suskind, E., Tannenbaum, S. and Suskind, D., 2014. Pilot testing of a parent-directed intervention (Project ASPIRE) for underserved children who are deaf or hard of hearing. Child Language Teaching and Therapy, 30(1), pp.91-102.

Seitz, S. and Marcus, S., 1976. Mother-child interactions: A foundation for language development. Exceptional children, 42(8), pp.445-449.

Simmons-Martin, A., 1981. Efficacy report: Early education project. Journal of the Division for Early Childhood, 4(1), pp.5-10.

Snoddon, K., 2009. American Sign Language and early literacy: Research as praxis. University of Toronto.

Steinberg, D.D., 1982. Overcoming linguistic limitations of hearing-impaired children through teaching written language. Topics in Language Disorders.

Störbeck, C. and Pittman, P., 2008. Early intervention in South Africa: Moving beyond hearing screening. International Journal of Audiology, 47(sup1), pp.S36-S43.

Suskind, D.L., Graf, E., Leffel, K.R., Hernandez, M.W., Suskind, E., Webber, R., Tannenbaum, S. and Nevins, M.E., 2016. Project ASPIRE: Spoken language intervention curriculum for parents of low-socioeconomic status and their Deaf and Hard-of-Hearing Children. Otology & Neurotology, 37(2), pp.e110-e117.

Suzuki, S. and Notoya, M., 1984. Teaching written language to deaf infants and preschoolers. Topics in early childhood special education, 3(4), pp.10-16.

Takala, M., Kuusela, J. and Takala, E.P., 2000. " A good future for deaf children": A five-year sign language intervention project. American Annals of the Deaf, pp.366-374.

Thompson, M.D. and Swisher, M.V., 1985. Acquiring language through total communication. Ear and hearing, 6(1), pp.29-32.

Vernon, M. and Koh, S.D., 1971. Effects of oral preschool compared to early manual communication on education and communication in deaf children. American Annals of the Deaf, pp.569-574.

Watkins, S., 1987. Long term effects of home intervention with hearing-impaired children. American Annals of the Deaf, 132(4), pp.267-271.

Watkins, S., Pittman, P. and Walden, B., 1998. The deaf mentor experimental project for young children who are deaf and their families. American Annals of the Deaf, 143(1), pp.29-34.
